# Pleiotropic regulation of a glucose-specific PTS in *Clostridium acetobutylicum* for high-efficient butanol production from corn stover without detoxification

**DOI:** 10.1186/s13068-019-1604-7

**Published:** 2019-11-07

**Authors:** Youduo Wu, Yidi Bai, Daojing Zhang, Chi Cheng, Lijie Chen, Fengwu Bai, Chuang Xue

**Affiliations:** 1 School of Bioengineering, Dalian University of Technology, No 2 Linggong Road, Dalian, 116024 China; 2 State Key Laboratory of Bioreactor Engineering, East China University of Science and Technology, Shanghai, 200237 China; 3 School of Life Sciences and Biotechnology, Shanghai Jiao Tong University, Shanghai, 200240 China

**Keywords:** *Clostridium acetobutylicum*, Corn stover, PTS regulation, Inhibitor tolerance, Butanol productivity

## Abstract

**Background:**

Corn stover (CS) is evaluated as the most favorable candidate feedstock for butanol production via microbial acetone–butanol–ethanol (ABE) fermentation by *Clostridium acetobutylicum*. By independent acid pretreatment and enzymatic hydrolysis, fermentable sugars (mainly glucose and xylose) were released, of which glucose was naturally utilized as the most preferred carbon source by *C. acetobutylicum*. However, the ABE fermentation using corn stover hydrolysate (CSH) without detoxification is typically limited to poor sugars utilization, butanol production and productivity. In the presence of pretreatment-derived inhibitors, the intracellular ATP and NADH, as important factors involved in cell growth, solventogenesis initiation and stress response, are exceedingly challenged owing to disrupted glucose phosphotransferase system (PTS). Therefore, there is a necessity to develop effective engineering approaches to overcome these limitations for high-efficient butanol production from CSH without detoxification.

**Results:**

PTS-engineered *C. acetobutylicum* strains were constructed via overexpression and knockout of gene *glcG* encoding glucose-specific PTS IICBA, which pleiotropically regulated glucose utilization, cell growth, solventogenesis and inhibitors tolerance. The PTS^GlcG^-overexpressing strain exhibited high fermentation efficiency, wherein butanol production and productivity was 11.1 g/L and 0.31 g/L/h, compared to those of 11.0 g/L and 0.15 g/L/h with the PTS^GlcG^-deficient strain. During CSH culture without detoxification, the PTS^GlcG^-overexpressing strain exhibited desirable inhibitors tolerance and solventogenesis with butanol production of 10.0 g/L, increased by 300% and 400% compared to those of 2.5 and 2.0 g/L with the control and PTS^GlcG^-deficient strains, respectively. As a result of extra glucose and 10 g/L CaCO_3_ addition into CSH, butanol production and productivity were further maximized to 12.5 g/L and 0.39 g/L/h. These validated improvements on the PTS^GlcG^-overexpressing strain were ascribed to not only efficient glucose transport but also its cascading effects on intracellular ATP and NADH generation, solventogenesis initiation and inhibitors tolerance at the exponential growth phase.

**Conclusion:**

The PTS^GluG^ regulation could be an effective engineering approach for high-efficient ABE fermentation from lignocellulosic hydrolysates without detoxification or wastewater generation, providing fundamental information for economically sustainable butanol production with high productivity.

## Background

Considering the worldwide depletion of fossil fuel resources, fluctuating crude oil prices as well as growing environmental concerns, sustainable butanol production via clostridial acetone–butanol–ethanol (ABE) fermentation is arousing intensive attention due to the unique physicochemical properties of butanol as a promising substitute for fossil-based energy [1–3]. Corn stover (CS) is well accepted as the most favorable candidate feedstock for sustainable ABE fermentation due to its widespread availability and high residue yield [4–6]. Followed by indispensable acid pretreatment and enzymatic hydrolysis, fermentable sugars (mainly glucose and xylose) are released from CS [4, 7], of which glucose is utilized as the most preferred carbon source. Although H_2_SO_4_-pretreatment is cost-effective and widely used for preparing corn stover hydrolysate (CSH) at industrial scale, the major drawback of this pretreatment process is the formation of degradation products such as weak acids, furan derivatives and phenolic compounds, which exhibit combined toxicities on clostridia [4, 7]. As a result, the fermentative process is severely inhibited without apparent sugar utilization, cell growth or solventogenesis, thus making butanol production from lignocellulosic feedstocks less sustainable and cost-competitive.

In recent years, numerous efforts have been made on development of pretreatment and detoxification methods, butanol recovery techniques, as well as strain reinforcements via mutagenesis, genetic manipulation and metabolic perturbations [8–17]. Particularly, significant improvements have been achieved with engineered solventogenic strains by either eliminating carbon catabolite repression or enhancing xylose metabolism. However, these genetically engineered strains were tested in synthetic media rather than detoxified lignocellulosic hydrolysates [11, 14–17]. Until now, of the best available strains for CS-based ABE fermentation, the highest ABE production and productivity of 26.3 g/L and 0.31 g/L/h were achieved with *C. beijerinckii* P260 using over-limed H_2_SO_4_-pretreated CSH, while no fermentation was observed using non-detoxified hydrolysate [18]. Despite physical, chemical or biological detoxification methods developed for eliminating the toxicities of inhibitors, the resulting wastewater emission, energy cost, sugar loss and low productivity remain hurdles for the economic viability and environmental sustainability at industrial scale [4, 7, 8, 19]. Therefore, there is a necessity to develop inhibitor-tolerating strains applicable to CSH culture without detoxification or wastewater generation.

When exposed to high levels of inhibitors like HMF and furfural, *C. acetobutylicum* cells are affected by uncoupling cellular functions associated with nutrient transport, cell replication, enzyme catalysis and stress response, resulting in inner accumulation of toxic metabolites and shortage of carbon, energy and reducing power [7]. In addition, ATP and NADH are drained during stress response such as heat shock proteins biosynthesis, efflux pumps and inhibitors transformation, thus shifting metabolism towards acid production to compensate for the decreased ATP supply [20]. Taken together, the significant issues for CHS culture lie in the disruption of glucose-specific PTS as well as ATP- and NADH-draining effects of microbial inhibitors [7]. More recently, micronutrient zinc as medium addictive affected ABE fermentation at metabolic and transcriptomic levels of *C. acetobutylicum* [21–23]. The upregulated expression of gene *glcG* encoding glucose-specific PTS IICBA led to efficient glucose transport, which evoked cascading effects on cofactors generation, solventogenesis initiation and stress tolerance [22]. Based on these findings, the objective of this work is to develop PTS^GlcG^-engineered *C. acetobutylicum* applicable to CSH culture without detoxification. Finally, PTS^GlcG^ overexpression exerted pleiotropic roles facilitating CSH culture, and thus could be an effective engineering approach for high-efficient butanol production from lignocellulosic feedstocks.

## Results and discussions

### Pleiotropic regulation of PTS^GluG^ on ABE fermentation

**Fig. 1:**
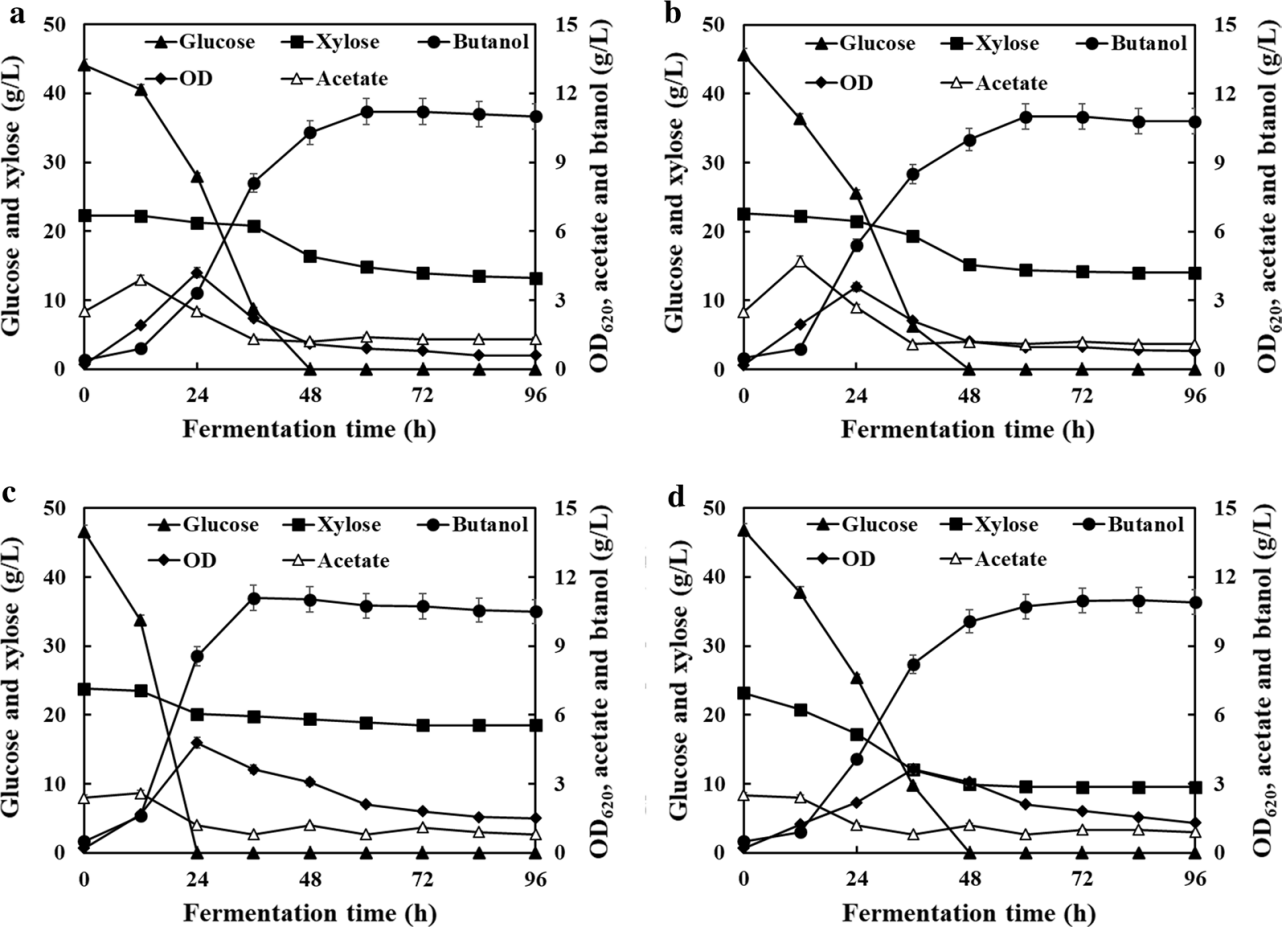
PTS^GlcG^-mediated regulation on ABE fermentation using glucose/xylose mixture as carbon sources by strains of **a** *C. acetobutylicum* L7; **b** *C. acetobutylicum* L7(pPthl); **c** *C. acetobutylicum* L7(GlcG); **d** *C. acetobutylicum* L7(ΔGlcG)

The mixture of glucose and xylose was first selected as carbon sources by the wild-type strain L7, control strain L7(pPthl), PTS^GlcG^-overexpressing strain L7(GlcG) and PTS^GlcG^-deficient strain L7(ΔGlcG). Comparative profiles and results were illustrated in Fig. 1 and Table 1, respectively. Similar to strain L7, 45.6 g/L glucose and 8.6 g/L xylose were slowly utilized by strain L7(pPthl) with butanol production and productivity of 11.0 g/L and 0.18 g/L/h. The acetate production achieved the highest level of 4.7 g/L at 12 h and then decreased to 0.8 g/L until the end of fermentation. As for strain L7(ΔGlcG), 46.8 g/L glucose and 13.7 g/L xylose were simultaneously consumed, by which xylose utilization was promoted while little difference was observed on glucose utilization, reaching butanol production and productivity of 11.0 g/L and 0.15 g/L/h. In addition, less than 2.5 g/L acetate was detected during the whole fermentation. These observations were identical to the results as recently reported [11], wherein disrupting PTS^GlcG^ facilitated simultaneous utilization of glucose, xylose and arabinose by *C. acetobutylicum*. During batch culture with strain L7(GlcG), 46.6 g/L glucose was rapidly depleted within 24 h, achieving an average consumption rate of 1.94 g/L/h, compared to those of 0.84 and 0.89 g/L/h from strains L7(pPthl) and L7(ΔGlcG), respectively. However, only 5.3 g/L xylose was utilized due to rapid butanol production up to 11.1 g/L and its severe toxicity on cells. Finally, butanol productivity of 0.31 g/L/h was greatly increased by 72.2% and 106.7% compared to those of 0.18 and 0.15 g/L/h from strains L7(pPthl) and L7(ΔGlcG), respectively. In particular, strain L7(GlcG) produced only 2.6 g/L acetate at 12 h and grew fast with a peak OD_620_ of 4.8 at 24 h, compared to that of 3.6 at 36 h for strain L7(ΔGlcG), indicating PTS^GlcG^-mediated pleiotropic roles in glucose/xylose utilization, exponential growth and solventogenesis initiation.

**Table 1:** Comparative results of batch ABE fermentation by *C. acetobutylicum* strains

Strain	Sugar utilized (g/L)	Products (g/L)					*Y*_B/ABE_ (g/g)	*P*_B/ABE_ (g/L/h)
		Acetate	Butyrate	Acetone	Butanol	Ethanol		
7% glucose/xylose (2:1) mixture as carbon sources								
L7	44.1 ± 1.1/9.1 ± 0.9	1.3 ± 0.2	1.0 ± 0.2	6.5 ± 0.4	11.2 ± 0.5	1.2 ± 0.1	0.21/0.36	0.19/0.32
L7(pPthl)	45.6 ± 1.5/8.6 ± 0.8	1.1 ± 0.1	0.9 ± 0.1	6.0 ± 0.3	11.0 ± 0.5	1.8 ± 0.1	0.20/0.35	0.18/0.31
L7(GlcG)	46.6 ± 1.3/5.3 ± 0.8	0.8 ± 0.1	0.5 ± 0.1	6.6 ± 0.5	11.1 ± 0.4	1.9 ± 0.2	0.21/0.38	0.31/0.54
L7(ΔGlcG)	46.8 ± 1.5/13.7 ± 1.1	0.9 ± 0.1	0.4 ± 0.1	6.1 ± 0.3	11.0 ± 0.5	1.2 ± 0.1	0.18/0.30	0.15/0.25
Non-detoxified H_2_SO_4_-pretreated CSH as carbon sources^a^								
L7	15.6 ± 1.3	2.4 ± 0.2	2.1 ± 0.3	1.8 ± 0.1	2.5 ± 0.2	0.5 ± 0.1	0.16/0.31	0.07/0.13
L7(pPthl)	15.2 ± 1.1	2.8 ± 0.2	2.3 ± 0.2	1.7 ± 0.1	2.5 ± 0.2	0.5 ± 0.1	0.16/0.31	0.07/0.13
L7(GlcG)	45.4 ± 1.6	1.3 ± 0.1	1.1 ± 0.1	6.0 ± 0.3	10.0 ± 0.4	1.3 ± 0.1	0.22/0.38	0.28/0.48
L7(ΔGlcG)	12.3 ± 1.4	1.7 ± 0.2	1.4 ± 0.2	1.2 ± 0.1	2.0 ± 0.1	0.4 ± 0.1	0.16/0.29	0.04/0.08
Non-detoxified enzymatically H_2_SO_4_-pretreated CSH as carbon sources								
L7	16.5 ± 1.3^b^	2.6 ± 0.2	2.2 ± 0.3	1.4 ± 0.1	3.0 ± 0.2	0.4 ± 0.1	0.18/0.29	0.08/0.13
L7(pPthl)	15.6 ± 1.0^b^	2.9 ± 0.3	2.3 ± 0.2	1.5 ± 0.1	2.7 ± 0.1	0.5 ± 0.1	0.17/0.30	0.08/0.13
L7(GlcG)	34.5 ± 1.9^b^	1.8 ± 0.2	1.5 ± 0.2	3.9 ± 0.2	7.2 ± 0.3	0.9 ± 0.1	0.21/0.35	0.20/0.33
	45.0 ± 1.5^c^	2.7 ± 0.3	2.1 ± 0.2	5.6 ± 0.3	10.0 ± 0.3	1.0 ± 0.1	0.22/0.37	0.28/0.46
	52.5 ± 2.0^d^	1.5 ± 0.1	1.2 ± 0.2	6.6 ± 0.4	11.0 ± 0.4	1.6 ± 0.1	0.21/0.37	0.34/0.60
	60.0 ± 1.8^e^	2.1 ± 0.2	1.7 ± 0.1	6.7 ± 0.4	12.5 ± 0.4	1.8 ± 0.2	0.21/0.35	0.39/0.66

^a^With glucose supplemented to 60 g/L total sugars

^b^CSH with 45 g/L total sugars and no nutrients added

^c^CSH with 45 g/L total sugars and 10 g/L CaCO_3_ added

^d^CSH with glucose supplemented to 60 g/L total sugars and no nutrients added

^e^CSH with glucose supplemented to 60 g/L total sugars and 10 g/L CaCO_3_ added

Especially for xylose consumption within the first 36 h of fermentation, only 2.5 and 3.2 g/L xylose were utilized by strains L7 and L7(pPthl), respectively, indicating that severe carbon catabolite repression on xylose consumption occurred in the presence of glucose. Finally, 6.6 and 5.4 g/L xylose were consumed by strains L7 and L7(pPthl) from 36 h to the end of fermentation. Similarly, as low as 3.7 g/L xylose was consumed within the first 24 h of fermentation by strain L7(GlcG). However, in the absence of glucose, only 1.6 g/L xylose was further consumed from 24 h to the end of fermentation due to the severe inhibition of high butanol concentration (> 9 g/L). Despite more xylose consumed rapidly by PTS^GlcG^-deficient strain within the first 48 h in the presence of glucose, the fermentative process remained inefficient due to low butanol yield and prolonged metabolism. Until recently, efficient glucose/xylose culture has been achieved by adding calcium carbonate and zinc sulfate, wherein butanol production and productivity were maximized to 13.9 g/L and 0.35 g/L/h [23]. Global transcriptomic and proteomic results showed that CaCO_3_ and ZnSO_4_ could act upon overall cellular functions associated with sugar utilization, central carbon metabolism and stress response at global levels [22, 24–26]. Particularly, followed by zinc addition, the expression of gene *glcG* was 3.62-fold upregulated at the exponential growth phase of *C. acetobutylicum*. Additionally, intracellular metabolites analysis showed that central carbon flux was earlier redistributed towards solventogenesis owing to increased supplies of ATP and NADH as driving forces [22]. Hence, fermentation efficiency depends on not only strains used but a rapid shift towards solventogenesis, which was mainly attributed to the coordination of metabolic cues and their cascading effects on *C. acetobutylicum*.

### Enhanced inhibitors tolerance during CSH culture without detoxification

**Fig. 2:**
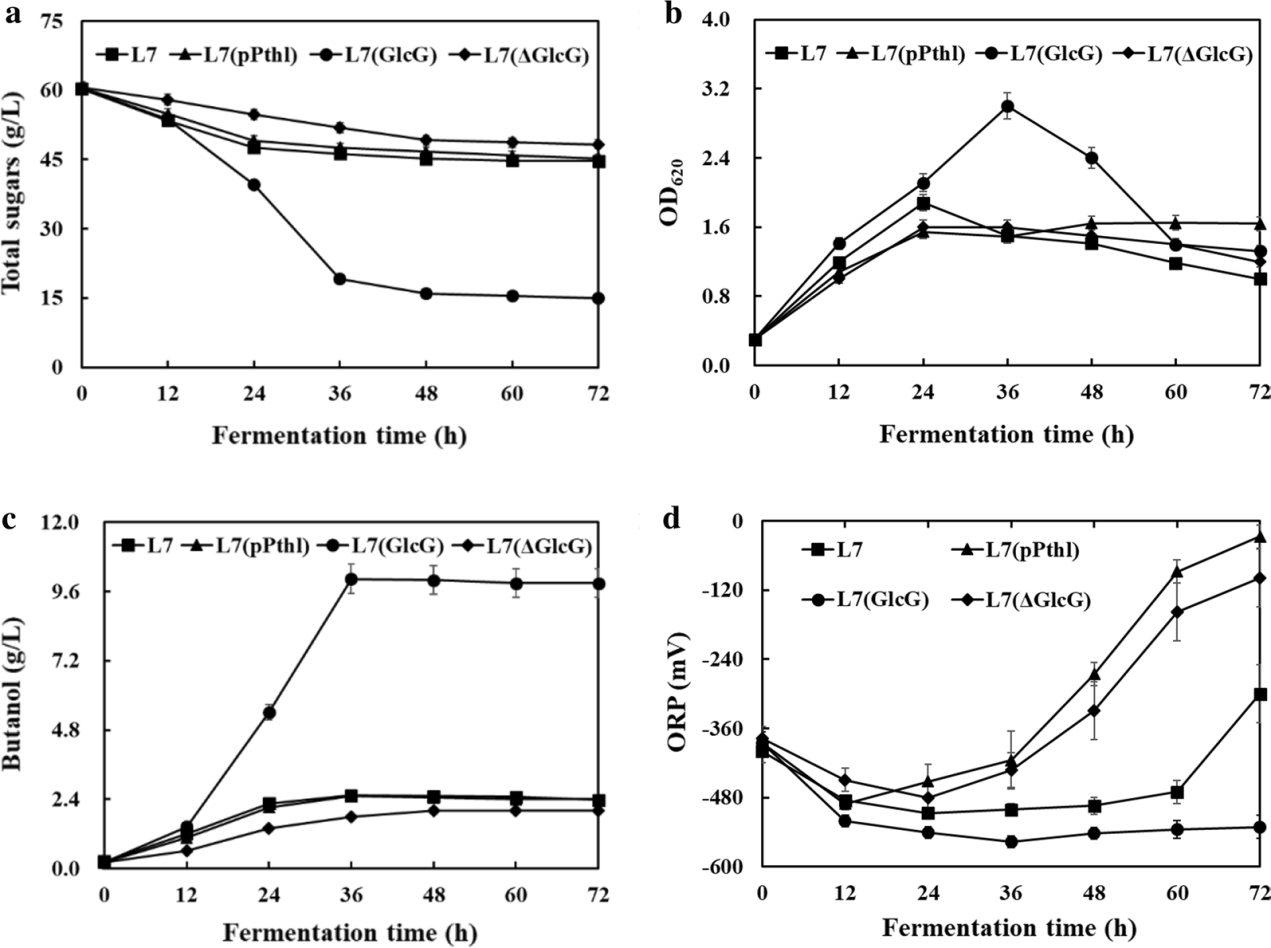
PTS^GlcG^-mediated impact on microbial inhibitors tolerance of *C. acetobutylicum* using non-detoxified H_2_SO_4_-pretreated CSH. Comparative fermentation profiles of **a** total sugars; **b** OD_620_; **c** butanol; **d** ORP

The basic chemical compositions of unpretreated CS solids were 40.8% cellulose, 15.2% hemicellulose, 32.1% acid insoluble lignin and ash. Followed by acid pretreatment, the pretreated CS solids were mainly composed of 56.5% cellulose, 4.5% hemicellulose, 34.7% acid insoluble lignin and ash. The recovery rates of solid, cellulose and hemicellulose were 57.8%, 79.5% and 17.1%, respectively. In the H_2_SO_4_-pretreated CSH, the initial concentration of total sugars was 22.0 g/L (3.0 g/L glucose, 15.5 g/L xylose and 3.5 g/L arabinose), and the initial concentrations of acetate, furfural and HMF as the major inhibitory compounds were 4.0, 1.5, and 0.9 g/L, respectively. After 10% inoculation, the concentration of total sugars was adjusted to ~ 60 g/L by adding glucose. As shown in Fig. 2 and Table 1, the regulatory impacts of PTS^GlcG^ on inhibitors tolerance were further investigated using the non-detoxified H_2_SO_4_-pretreated CSH. Similar to strain L7, strain L7(pPthl) exhibited poor cell growth and solventogenesis, wherein 2.5 g/L butanol was produced from 15.2 g/L total sugars (mostly glucose) with an average sugar consumption rate of 0.42 g/L/h. The residual acetate decreased to 2.8 g/L. As for strain L7(ΔGlcG), only 12.3 g/L total sugars (8.8 g/L glucose and 3.5 g/L xylose) were utilized with an average sugar consumption rate of 0.34 g/L/h, resulting in slightly decreased butanol production of 2.0 g/L. The residual acetate decreased to 1.7 g/L. It should be noted that 1.3 g/L furfural and 0.8 g/L HMF remained in the fermentation broth, implying poor metabolic transformation of furfural and HMF by strains L7(pPthl) and L7(ΔGlcG).

Particularly for strain L7(GlcG), as high as 45.4 g/L total sugars (42.7 g/L glucose and 2.7 g/L xylose) was rapidly utilized within 36 h, reaching a peak OD_620_ of 3.0 and an average sugar consumption rate of 1.26 g/L/h. Finally, butanol production of 10.0 g/L was achieved together with yield and productivity of 0.22 g/g and 0.28 g/L/h, respectively, which increased by 300%, 37.5% and 300% compared to those of strain L7(pPthl). More strikingly, the residual acetate, furfural and HMF significantly decreased to 1.3, 0.3 and 0.1 g/L, respectively, demonstrating that these pretreatment-derived inhibitors could be largely transformed into less toxic compounds by strain L7(GlcG), thus making the non-detoxified CSH culture sustainable. For example, the oxidation–reduction potential (ORP) was maintained at the range of − 550 to − 520 mV, which was consistent with the active cellular metabolisms by PTS^GlcG^ overexpression. Correspondingly, the ORP increased rapidly after 24 h culture with both strains L7(pPthl) and L7(ΔGlcG), wherein cell death and premature termination occurred owing to their insufficient supports on carbon, energy and reducing power. Until now, the highest butanol production of 14.5 g/L was achieved by *C. beijerinckii* P260 from detoxified H_2_SO_4_-pretreated CSH (60.3 g/L total sugars by adding glucose) [18], however, butanol productivity of 0.17 was much lower than that of 0.28 g/L/h in non-detoxified CSH culture by strain L7(GlcG).

Microbial inhibitors such as furfural and 5-hydroxymethyl furfural (HMF) could be naturally transformed into less inhibitory products via the NADH-dependent metabolic detoxification process in *Clostridium* strains [7], which in turn hinders a metabolic shift towards solventogenesis due to excess NADH consumption in *C. acetobutylicum* [27]. Furthermore, the decreased ATP supply impairs cell growth and stress response, including membrane modification, heat shock proteins biosynthesis and efflux pumps. Therefore, boosting intracellular ATP and NADH supplies has been validated as a rational approach for combating these negative effects of inhibitors [28–31]. For instance, glycerol as a co-substrate with glucose (2:1, mol/mol) could generate additional ATP and NADH and exert positive effects on cell growth, alcohol/aldehyde dehydrogenases activities and furfural/HMF transformation [12]. When subjected to 5 g/L furfural during ABE fermentation, glycerol generated a 1.8-fold increase in NADH level, which accounted for 2.3-fold increase in furfural detoxification rate, glucose utilization and butanol production compared to the control without glycerol addition [12]. However, the naturally poor glycerol utilization remains a bottleneck for most solventogenic clostridia strains, leading to unsustainable ATP/NADH support as well as unnecessary waste [32, 33]. Therefore, the demonstrated PTS^GlcG^ overexpression possibly boosted intracellular ATP/NADH supplies and evoked cascading effects on inhibitors tolerance.

### Increased intracellular ATP and NADH supplies by overexpressing PTS^GlcG^

**Fig. 3:**
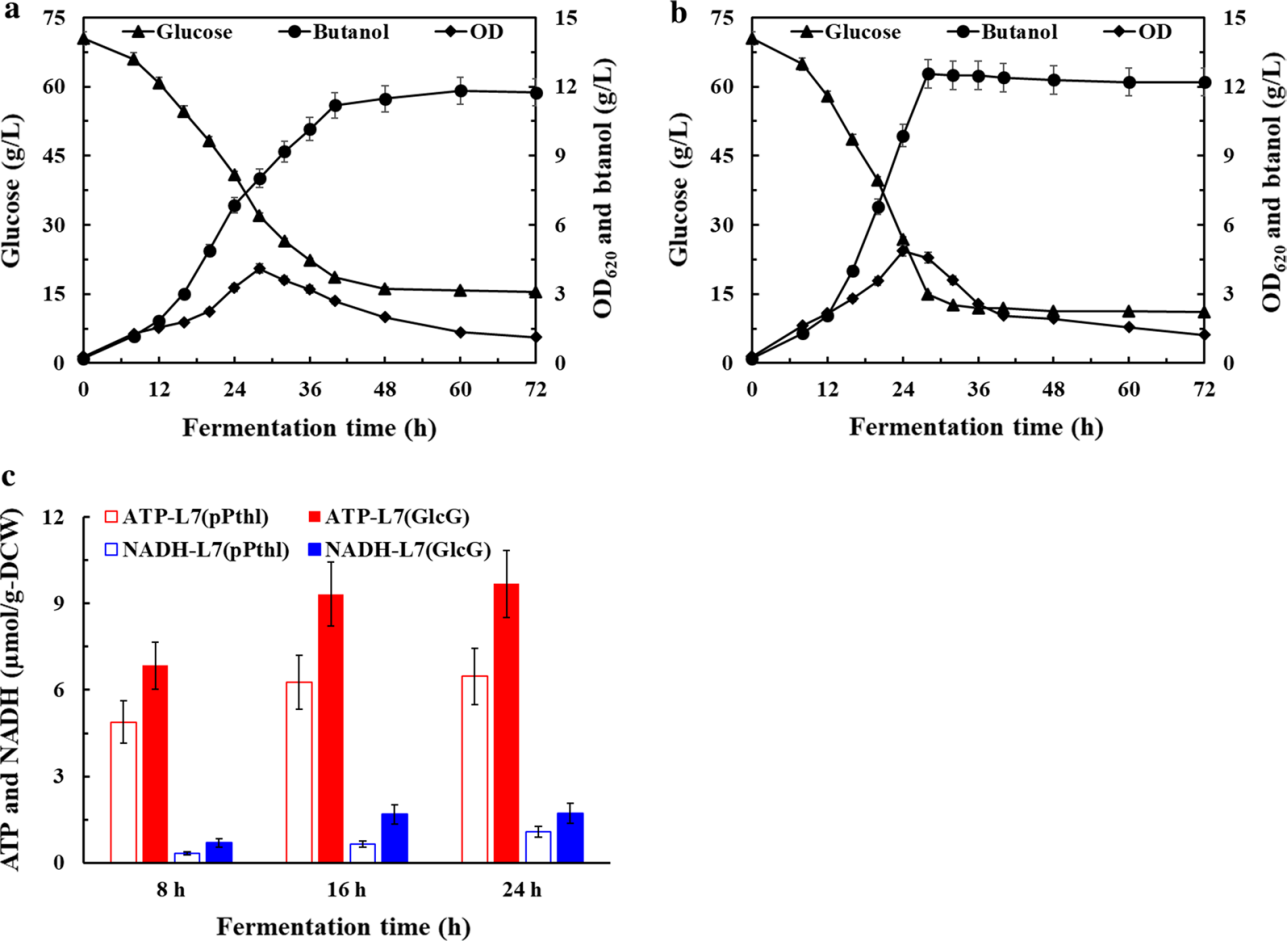
PTS^GlcG^-mediated effects on ABE fermentation using glucose as sole carbon source. Comparative profiles of **a** glucose, OD_620_ and butanol of strain L7(pPthl); **b** glucose, OD_620_ and butanol of strain L7(GlcG); **c** ATP and NADH of strains L7(pPthl) and L7(GlcG)

**Fig. 4:**
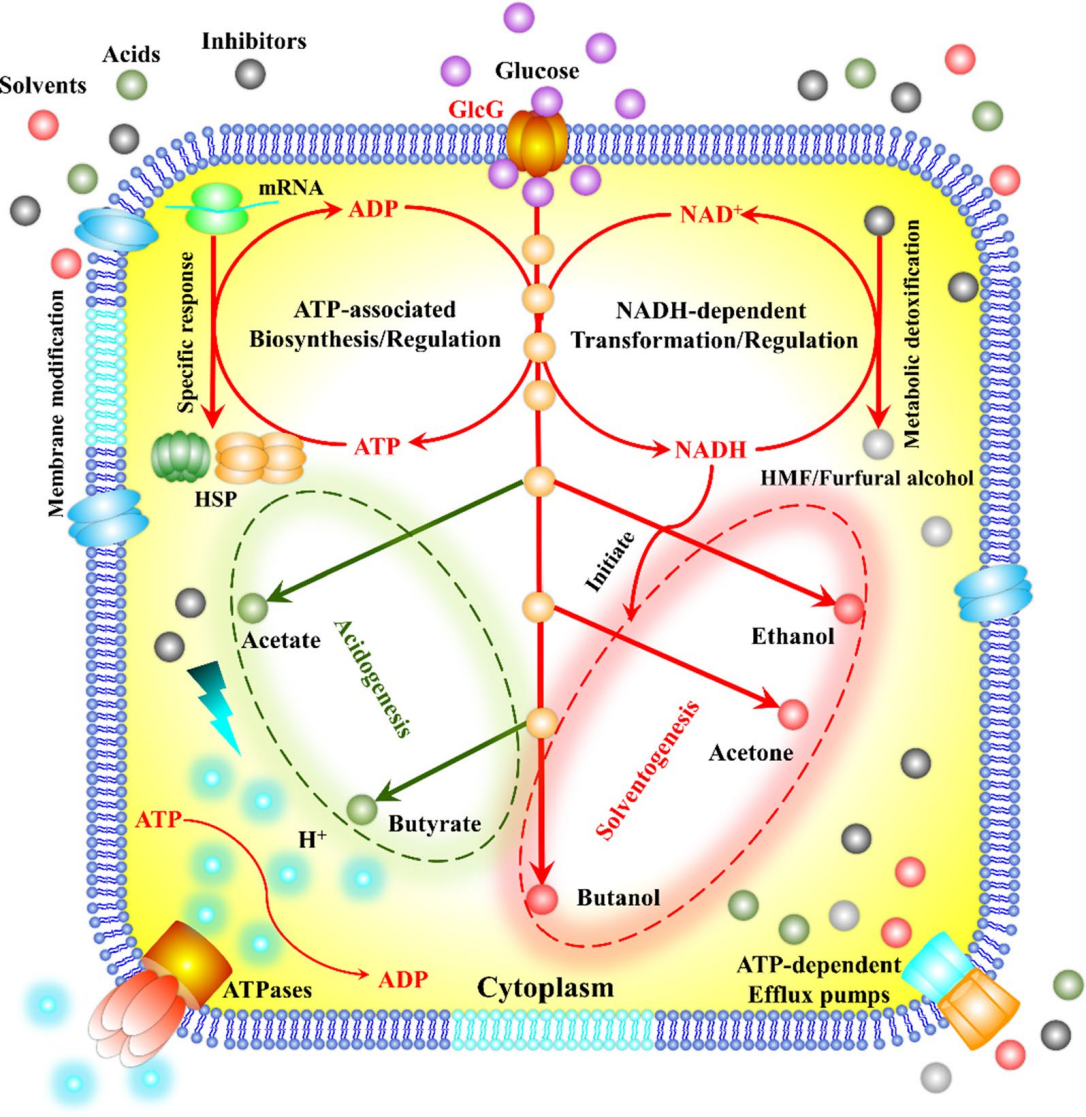
Pleiotropic regulation of PTS^GlcG^ overexpression on *Clostridium acetobutylicum*

For the sake of better understanding the intracellular energy and reducing power changes in *C. acetobutylicum* after PTS^GlcG^ overexpression, glucose-based batch culture was performed to analyze ATP and NADH levels of strains L7(pPthl) and L7(GlcG), respectively. As shown in Fig. 3, strain L7(GlcG) exhibited efficient glucose utilization and earlier solventogenesis, wherein 12.6 g/L butanol was achieved from 59.4 g/L glucose within only 28 h. Therefore, as high as 2.12 and 0.45 g/L/h of glucose consumption rate and butanol productivity were also achieved, respectively, which increased by 130.4% and 125% compared to those of strain L7(pPthl). Furthermore, the exponential growth of strain L7(GlcG) was significantly enhanced with a peak OD_620_ of 4.9 obtained at 24 h, compared to that of 4.1 obtained at 28 h for strain L7(pPthl). Since strain L7(GlcG) exhibited higher fermentation efficiency than strain L7(pPthl), the sampling times were 8, 16 and 24 h of the exponential growth phase for both strains. At the three sampling times of 8, 16 and 24 h, the ATP levels of strain L7(GlcG) were 6.85, 9.33 and 9.69 µmol/g-DCW, respectively, which increased by 40.4%, 49.0% and 49.8% compared to those of strain L7(pPthl), thus leading to 23.1%, 55.6% and 48.5% increase on the OD_620_. More importantly, the NADH levels of strain L7(GlcG) were 0.69, 1.68 and 1.73 µmol/g-DCW, respectively, which increased by 52.2%, 61.9% and 37.6% compared to those of strain L7(pPthl), which contributed to earlier solventogenesis initiation and NADH-driven inhibitors transformation. Therefore, as illustrated in Fig. 4, the experimental results demonstrated that overexpressing PTS^GlcG^ played pleiotropic roles regulating glucose transport and its cascading effects on cofactors generation, solventogenesis initiation and inhibitors tolerance of *C. acetobutylicum*.

### High-efficient ABE fermentation from non-detoxified CSH

**Fig. 5:**
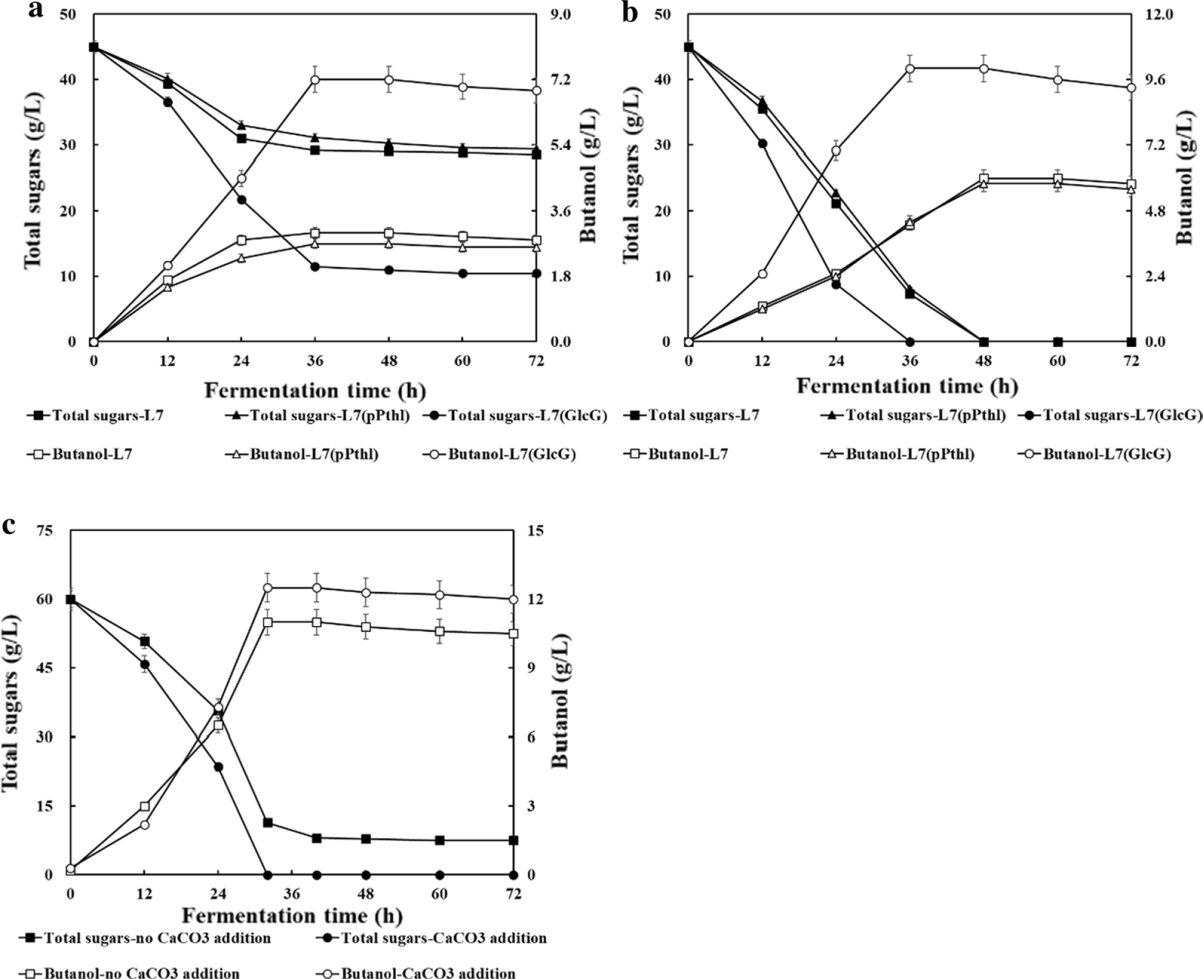
Batch ABE fermentation using non-detoxified enzymatically H_2_SO_4_-pretreated CSH. Fermentation conditions of **a** 45 g/L total sugars; **b** 45 g/L total sugars with 10 g/L CaCO_3_ addition; **c** 60 g/L total sugars with/without 10 g/L CaCO_3_ addition

To achieve cost-effective lignocellulosic butanol production, the enzymatically H_2_SO_4_-pretreated CSH was directly utilized for batch culture without detoxification (Fig. 5). Similarly, strains L7 and L7(pPthl) both exhibited poor fermentation performance and produced less than 3 g/L butanol at 36 h. As for strain L7(GlcG), 7.2 g/L butanol was produced from 34.5 g/L total sugars within 36 h. However, as high as 10.5 g/L residual sugars (mainly xylose) remained in the fermentation broth. As a result of 10 g/L CaCO_3_ addition, all sugars were rapidly depleted by strain L7(GlcG) within 36 h, wherein butanol production and productivity were further maximized to 10.0 g/L and 0.28 g/L/h. It should be noted that, under the same culture condition, butanol production was still limited to ~ 6.0 g/L with strains L7 and L7(pPthl), respectively, implying potential redistribution of central carbon flux towards butanol biosynthesis by overexpressing PTS^GlcG^. Actually, 100 g dry corn stover yielded ~ 45 g fermentable sugars (27.8 g glucose, ~ 14 g xylose and ~ 3.2 g arabinose) in this study. Given the fact that as high as ~ 66 g fermentable sugars could be extracted from 100 g dry CS [4], more fermentable sugars (especially glucose) could be released from pretreated corn stover if possible. Therefore, the high concentration of total sugars was further adjusted to 60 g/L by adding glucose after 10% inoculation. As expected, 60 g/L total sugars were all depleted by strain L7(GlcG) in the presence of only 10 g/L CaCO_3_. Butanol production and productivity of 12.5 g/L and 0.39 g/L/h could be achieved, which increased by 316.7% and 387.5% compared to those of strain L7(pPthl), making the detoxification process, wastewater generation and medium optimization unnecessary for non-detoxified CSH culture.

As summarized in Table 2, despite considerable efforts on improving CS-based ABE fermentation [10, 18, 34–36], high-efficient CSH culture remains a significant issue in synthetical consideration of sugar utilization, butanol production and productivity. For instance, Qureshi et al. reported that the highest butanol production of 14.5 g/L was achieved with productivity of 0.17 g/L/h by *C. beijerinckii* P260 using detoxified H_2_SO_4_-pretreated CSH [18]. Zhang et al. reported that 7.1 g/L butanol was produced with much lower productivity of 0.10 g/L/h from detoxified CSH by *C. acetobutylicum* ATCC 824 [36]. Until recently, butanol productivity was improved to 0.19 g/L/h using detoxified enzymatically NaOH-pretreated CSH by *C. beijerinckii* CC101 [37]. As reported by Gao et al., the butanol-tolerant *C. acetobutylicum* strain 206 was screened by NTG mutagenesis and proven to be robust for lignocellulosic ABE fermentation, wherein butanol productivity up to 0.14 g/L/h could be achieved using non-detoxified enzymatically NaOH-pretreated CSH [38]. However, no solventogenic clostridia strains to date are available for high-efficient CSH culture. Therefore, the demonstrated PTS^GlcG^ overexpression in this study was validated as a feasible engineering approach for high-efficient butanol production from lignocellulosic feedstocks without detoxification or wastewater generation.

**Table 2:** Comparisons of batch ABE fermentation using detoxified or non-detoxified CSH

Strain	Pretreatment method	Detoxification method	Products			References
			Butanol/ABE (g/L)	Yield (g/g)	Productivity (g/L/h)	
*C. acetobutylicum* ATCC 824	Alkaline twin-screw extrusion	Washing	7.1/11.2	0.18/0.29	0.10/0.16	Zhang et al. [36]
*C. acetobutylicum* ATCC 824	Steam explosion	Activated charcoal	NA/12.4	NA/0.30	NA/0.17	Wang and Chen [35]
*C. beijerinckii* P260	1% (v/v) H_2_SO_4_	Overliming	14.5/26.3	0.24/0.44	0.17/0.31	Qureshi et al. [18]
*C. beijerinckii* P260	1% (v/v) H_2_SO_4_	Overliming	NA/24.4	NA/0.44	NA/0.30	Qureshi et al. [34]
*C. beijerinckii* CC101	2% (v/v) NaOH	Washing	11.2/19.8	0.28/0.49	0.19/0.33	Xue et al. [37]
*C. acetobutylicum* ATCC 824	Steam explosion	ND	2.2/3.7	NA/NA	0.03/0.05	Wang and Chen [35]
*C. acetobutylicum* strain 206	2% (v/v) NaOH	ND	9.8/15.4	0.26/0.41	0.14/0.21	Gao et al. [38]
*C. beijerinckii* P260	1% (v/v) H_2_SO_4_	ND	No fermentation			Qureshi et al. [18]
*C. acetobutylicum* L7	1% (v/v) H_2_SO_4_	ND	3.0/4.8	0.18/0.29	0.08/0.13	This study
*C. acetobutylicum* L7(GlcG)	1% (v/v) H_2_SO_4_	ND	12.5/21.0	0.21/0.35	0.39/0.66	This study

*NA* not available, *ND* not detoxified

## Conclusions

In this study, PTS^GlcG^-engineered *C. acetobutylicum* strains were constructed via gene *glcG* overexpression and knockout to address the major problems during non-detoxified CSH culture. The PTS^GlcG^-overexpressing strain exhibited remarkable improvements in glucose utilization, exponential growth and inhibitors tolerance, which was ascribed to efficient glucose transport but also its cascading effects on intracellular ATP and NADH generation, solventogenesis initiation and inhibitors tolerance. Finally, 12.5 g/L butanol was achieved within 32 h of non-detoxified CSH culture, resulting in significantly improved butanol productivity up to 0.39 g/L/h, and thus making butanol production from lignocellulosic feedstocks more economically sustainable and environmentally friendly.

## Materials and methods

### Bacterial strains, primers and plasmids

All bacterial strains and plasmids used in this study are listed in Table 3. Generally, strain *C. acetobutylicum* L7, adapted from the wild-type strain *C. acetobutylicum* ATCC 824 as previously reported [21], was used for constructing the control and PTS-engineered strains. Strains *E. coli* DH5α and DH10B cultured with LB medium or agar containing 10 μg/mL ampicillin and 50 μg/mL spectinomycin were used for plasmids amplification and in vivo methylation. Based on the *C. acetobutylicum* ATCC 824 genome, gene *glcG* synthesized by Sangon (Shanghai, China) was digested with the *SalI*/*KpnI* restriction sites and then ligated into the control plasmid pIMP1-P_thl_, yielding the target plasmid pIMP1-P_thl_-GlcG. Plasmids pIMP1-P_thl_ and pIMP1-P_thl_-GlcG were first methylated in *E. coli* DH10B and then electroporated into strain L7, respectively, according to the standard protocols [39]. The resulting cells were then cultured on RCM (Thermo Fisher, Oxoid Ltd.) agar containing 50 μg/mL erythromycin for single colony selection, yielding the control strain L7(pPthl) and PTS^GlcG^-overexpressing strain L7(GlcG). According to the method described by Xiao et al., the PTS^GlcG^-deficient strain L7(ΔGlcG) was constructed via intron-mediated knockout system with an intron inserted at 269/270 bp of gene *glcG* [11], and all the related primers were synthesized by Sangon (see Additional file 1: Table S1). All the commercial enzymes were purchased from New England Biolabs (Beverly, MA).

**Table 3:** Bacterial strains and plasmids used in this study

Strains/plasmids	Relevant characteristics	Source/references
Bacterial strains		
*E. coli*		
DH5α	Host cells for gene cloning and plasmids amplification	Invitrogen
DH10B	Strain used to methylate the vector	Invitrogen
*C. acetobutylicum*		
ATCC 824	Wild-type strain	ATCC
L7	Adapted from *C. acetobutylicum* ATCC 824	[21]
L7(pPthl)	Control strain, L7 containing the control plasmid pIMP1-Pthl	This study
L7(GlcG)	L7 containing the plasmid pIMP1-Pthl-glcG	This study
L7(ΔGlcG)	PTS^GlcG^-deficient strain via intron-mediated knockout	This study
Plasmids		
pAN1	Φ3TI, p15A origin; *Spe*^*r*^	[39]
PSY6	Group II intron, ltrA	[41]
pIMP1	*Amp*^*r*^; *MLS*^*r*^; repL, ColE1 origin, shuttle vector	[11]
pIMP1-Pthl	Control vector carrying *thl* promoter, derived from pIMP1	[11]
pIMP1-Pthl-glcG	Derived from pIMP1-Pthl with *glcG* overexpression in L7	This study
PSY-GlcG	Vector for intron insertion at 269/270 bp of *glcG* in L7	This study

### Acid pretreatment of corn stover

The CS (Pioneer variety) used in this study was obtained from a local farmer in Shandong and milled into 1–2 mm particles using a hammer mill. As documented by Malmierca et al., one hundred grams of milled CS was soaked with 1 L 1% (v/v) dilute H_2_SO_4_ solution in a stirred tank at 121 °C for 90 min, followed by cooling to room temperature and adjusting pH to 5.0 using 10 M NaOH [40]. The initial concentration of total sugars in the H_2_SO_4_-pretreated CSH was 22 g/L, which was further added to 60 g/L with extra glucose when necessary.

### Enzymatic hydrolysis of acid-pretreated corn stover

As optimized by Xue et al., the resulting H_2_SO_4_-pretreated CS mixture containing 30 mM citrate was hydrolyzed using cellulase (Tianjin Novozymes Biotechnology, China) with 20 FPU/g-CS at 50 °C, pH 4.8 and 200 rpm for 72 h [37]. The enzymatically H_2_SO_4_-pretreated CSH was obtained by centrifugation at 8000×*g* for 5 min to remove sediments, and then stored at 4 °C for subsequent batch culture without detoxification process or medium optimization. Followed by 10% inoculation during CSH culture, the initial concentration of total sugars was ~ 45 g/L, which was added to 60 g/L with extra glucose when necessary.

### Media and culture conditions

The media for pre-culture and seed culture were as previously described [21, 23]. The standard fermentation medium is composed of (g/L): glucose or glucose/xylose mixture (2:1, w/w) 70.0, yeast extract 2.0, K_2_HPO_4_ 0.50, KH_2_PO_4_ 0.50, MgSO_4_·7H_2_O 0.20, MnSO_4_·H_2_O 0.10, FeSO_4_·7H_2_O 0.01, CH_3_COONH_4_ 3.22, para-amino-benzoic acid 0.01 and biotin 0.01. The CSHs mentioned above were directly utilized for batch ABE fermentation, respectively. All CSHs and media were sterilized at 121 °C for 15 min, followed by cooling to room temperature and added with 10 μg/mL erythromycin when necessary. The initial pH for batch culture was adjusted to 5.5 using 3 M H_2_SO_4_ or 3 M NaOH after 10% inoculation. All chemical reagents used in this study were of analytical grade or equivalent and purchased from Sangon.

### Batch ABE fermentation

Batch ABE fermentation was carried out in a stirred tank containing CSH or standard medium under anaerobic conditions as previously described [21]. After 10% inoculation, the initial fermentation pH was adjusted to 5.5 using 3 M H_2_SO_4_ or NaOH. All the experiments were triplicated and samples were taken for analyzing cell growth, residual sugar(s), acids and ABE production. Particularly, intracellular ATP and NADH were quantified during batch culture using non-detoxified H_2_SO_4_-pretreated CSH.

### Analysis of intracellular ATP and NADH

During batch culture using glucose as the sole carbon source, *C. acetobutylicum* cells were collected at 8, 16 and 24 h by centrifugation at 10,000×*g* for 3 min at − 10 °C. The resulting cell pellets were quenched immediately with 500 μL solution mixture of methanol, acetonitrile and water (40:40:20, v/v, − 40 °C), and then frozen in liquid nitrogen for preparing crude extracts. According to our previous study [22], LC–MS/MS analysis was conducted for ATP quantification with an ACCELA HPLC system (Thermo Scientific, CA) equipped with an XBridge BEH Amide column (100 mm × 2.1 mm I.D., 2.5 μm, Waters, Ireland). Mass monitoring was achieved using a TSQ Quantum Ultra triple quadrupole mass analyzer (Thermo Scientific, CA) equipped with a heated electrospray ionization source (HESI). NADH assay was performed using a commercial kit (Sigma, MO). Cell pellets were first lysed using a Qiagen Tissue Lyser LT (Qiagen, Germany) at 50 oscillations/s for 3 min in the NADH extraction buffers (Sigma, MO), the resulting lysate was then used for NADH quantification at 450 nm with an iMark™ microplate reader (Bio-Rad, CA).

### Analytical methods

As previously described, cell growth was measured at 620 nm using a spectrophotometer (Thermo Spectronic, USA) [22]. The chemical compositions of unpretreated and pretreated CS solids were analyzed according to Laboratory Analytical Procedures [19].

The total sugars in CSH were determined by 3,5-dinitrosalicylic acid (DNS) method at 540 nm using the spectrophotometer. ABE were determined by a gas chromatography (Agilgent 6890A GC). Sugars (glucose, xylose and arabinose), acids (acetate and butyrate) and major inhibitors (furfural and HMF) were analyzed by a high-performance liquid chromatography (Waters 1525 HPLC). All the standard chemicals of sugars, acids and ABE were of quality HPLC gradient grade and purchased from Sigma-Aldrich (Saint-Louis, Missouri, USA). The butanol or ABE yield (*Y*_B/ABE_) was calculated as total butanol or ABE produced divided by the total sugars used and is expressed in g/g. The butanol or ABE productivity (*P*_B/ABE_) was calculated as total butanol or ABE produced divided by the fermentation time used and is expressed in g/L/h.

## Supplementary information

**Additional file 1: Table S1.** Primers used in this study.

## Data Availability

All data generated or analyzed in this study are included in this article and its Additional information files. The datasets used and/or analyzed during the current study are available from the corresponding author on reasonable request.

## Abbreviations

CSH: Corn stover hydrolysate
PTS: Phosphotransferase system
ABE: Acetone–butanol–ethanol
RCM: Reinforced *Clostridium* medium
ATP: Adenosine triphosphate
NADH: Nicotinamide adenine dinucleotide

## References

[1] Xue C, Zhao XQ, Liu CG (2013). Prospective and development of butanol as an advanced biofuel. Biotechnol Adv.

[2] Xue C, Zhao JB, Chen LJ, Yang ST, Bai FW (2017). Recent advances and state-of-the-art strategies in strain and process engineering for biobutanol production by *Clostridium acetobutylicum*. Biotechnol Adv.

[3] Ranjan A, Moholkar VS (2012). Biobutanol: science, engineering, and economics. Int J Energy Res..

[4] Baral NR, Slutzky JL, Shah A (2016). Acetone-butanol-ethanol fermentation of corn stover: current production methods, economic viability, and commercial use. FEMS Microbiol Lett..

[5] Gottumukkala LD, Haigh K, Görgens J (2017). Trends and advances in conversion of lignocellulosic biomass to biobutanol: microbes, bioprocesses and industrial viability. Renew Sust Energy Rev..

[6] Li J, Baral NR, Jha AK (2013). Acetone-butanol-ethanol fermentation of corn stover by *Clostridium* species: present status and future perspectives. World J Microbiol Biotechnol.

[7] Baral NR, Shah A (2014). Microbial inhibitors: formation and effects on acetone-butanol-ethanol fermentation of lignocellulosic biomass. Appl Microbiol Biotechnol.

[8] Jönsson LJ, Martín C (2016). Pretreatment of lignocellulose: formation of inhibitory by-products and strategies for minimizing their effects. Bioresour Technol.

[9] Yang ST, Zhao JB. Adaptive engineering of *Clostridium* for increased butanol production. 2013; US Patent 8450093.

[10] Guo T, Tang Y, Zhang QY (2012). *Clostridium beijerinckii* mutant with high inhibitor tolerance obtained by low-energy ion implantation. J Ind Microbiol Biotechnol.

[11] Xiao H, Gu Y, Ning YY (2011). Confirmation and elimination of xylose metabolism bottlenecks in glucose phosphoenolpyruvate-dependent phosphotransferase system-deficient *Clostridium acetobutylicum* for simultaneous utilization of glucose, xylose, and arabinose. Appl Environ Microb..

[12] Ujor V, Agu CV, Gopalan V (2014). Glycerol supplementation of the growth medium enhances in situ detoxification of furfural by *Clostridium beijerinckii* during butanol fermentation. Appl Microbiol Biotechnol.

[13] Grimmler C, Held C, Liebl W (2010). Transcriptional analysis of catabolite repression in *Clostridium acetobutylicum* growing on mixtures of d-glucose and d-xylose. J Biotechnol.

[14] Ren C, Gu Y, Hu S (2010). Identification and inactivation of pleiotropic regulator CcpA to eliminate glucose repression of xylose utilization in *Clostridium acetobutylicum*. Metab Eng.

[15] Jin L, Zhang H, Chen L (2014). Combined overexpression of genes involved in pentose phosphate pathway enables enhanced d-xylose utilization by *Clostridium acetobutylicum*. J Biotechnol.

[16] Gu Y, Li J, Zhang L (2009). Improvement of xylose utilization in *Clostridium acetobutylicum* via expression of the *talA* gene encoding transaldolase from *Escherichia coli*. J Biotechnol.

[17] Gu Y, Ding Y, Ren C (2010). Reconstruction of xylose utilization pathway and regulons in *Firmicutes*. BMC Genomics..

[18] Qureshi N, Saha BC, Hector RE (2010). Production of butanol (a biofuel) from agricultural residues: part II—use of corn stover and switchgrass hydrolysates. Biomass Bioenergy.

[19] Xue C, Zhang XT, Wang JF (2017). The advanced strategy for enhancing biobutanol production and high-efficient product recovery with reduced wastewater generation. Biotechnol Biofuels.

[20] Gheshlaghi R, Scharer JM, Moo-Young M (2009). Metabolic pathways of clostridia for producing butanol. Biotechnol Adv.

[21] Wu YD, Xue C, Chen LJ (2013). Effect of zinc supplementation on acetone-butanol-ethanol fermentation by *Clostridium acetobutylicum*. J Biotechnol.

[22] Wu YD, Xue C, Chen LJ (2015). Transcriptional analysis of micronutrient zinc-associated response for enhanced carbohydrate utilization and earlier solventogenesis in *Clostridium acetobutylicum*. Sci Rep..

[23] Wu YD, Xue C, Chen LJ (2016). Synergistic effect of calcium and zinc on glucose/xylose utilization and butanol tolerance of *Clostridium acetobutylicum*. FEMS Microbiol Lett..

[24] Han B, Ujor V, Lai LB (2013). Use of proteomic analysis to elucidate the role of calcium on acetone-butanol-ethanol (ABE) fermentation in *Clostridium beijerinckii* NCIMB 8052. Appl Environ Microbiol.

[25] Richmond C, Han B, Ezeji TC (2011). Stimulatory effects of calcium carbonate on butanol production by solventogenic *Clostridium* species. Cont J Microbiol..

[26] El Kanouni A, Zerdani I, Zaafa S (1998). The improvement of glucose/xylose fermentation by *Clostridium acetobutylicum* using calcium carbonate. World J Microbiol Biotechnol.

[27] Zhang Y, Han B, Ezeji TC (2012). Biotransformation of furfural and 5-hydroxymethyl furfural (HMF) by *Clostridium acetobutylicum* ATCC 824 during butanol fermentation. N Biotechnol.

[28] Ventura JR, Hu H, Jahng D (2013). Enhanced butanol production in *Clostridium acetobutylicum* ATCC 824 by double overexpression of 6-phosphofructokinase and pyruvate kinase genes. Appl Microbiol Biotechnol.

[29] Liu D, Yang Z, Wang P (2018). Towards acetone-uncoupled biofuels production in solventogenic *Clostridium* through reducing power conservation. Metab Eng.

[30] Agu CV, Ujor V, Ezeji TC (2019). Metabolic engineering of *Clostridium beijerinckii* to improve glycerol metabolism and furfural tolerance. Biotechnol Biofuels.

[31] Liu J, Guo T, Wang D (2016). Enhanced butanol production by increasing NADH and ATP levels in *Clostridium beijerinckii* NCIMB 8052 by insertional inactivation of Cbei_4110. Appl Microbiol Biotechnol.

[32] Girbal L, Croux C, Vasconcelos I (1995). Regulation of metabolic shifts in *Clostridium acetobutylicum* ATCC 824. FEMS Microbiol Rev.

[33] Vasconcelos I, Girbal L, Soucaille P (1994). Regulation of carbon and electron flow in *Clostridium acetobutylicum* grown in chemostat culture at neutral pH on mixtures of glucose and glycerol. J Bacteriol.

[34] Qureshi N, Cotta MA, Saha BC (2014). Bioconversion of barley straw and corn stover tobutanol (a biofuel) in integrated fermentation and simultaneous product recovery bioreactors. Food Bioprod Process.

[35] Wang L, Chen HZ (2011). Increased fermentability of enzymatically hydrolyzed steam-exploded corn stover for butanol production by removal of fermentation inhibitors. Process Biochem.

[36] Zhang YD, Hou TG, Li B (2014). Acetone-butanol-ethanol production from corn stover pretreated by alkaline twin-screw extrusion pretreatment. Bioprocess Biosyst Eng..

[37] Xue C, Wang ZX, Wang SD (2016). The vital role of citrate buffer in acetone-butanol-ethanol (ABE) fermentation using corn stover and high-efficient product recovery by vapor stripping-vapor permeation (VSVP) process. Biotechnol Biofuels.

[38] Gao K, Li Y, Tian S (2012). Screening and characteristics of a butanol-tolerant strain and butanol production from enzymatic hydrolysate of NaOH-pretreated corn stover. World J Microbial Biotechnol.

[39] Mermelstein LD, Papoutsakis ET (1993). In vivo methylation in *Escherichia coli* by the *Bacillus subtilis* phage phi 3T I methyltransferase to protect plasmids from restriction upon transformation of *Clostridium acetobutylicum* ATCC 824. Appl Environ Microbiol.

[40] Malmierca S, Díez-Antolínez R, Paniagua AI (2017). Technoeconomic study of biobutanol AB production. 1. Biomass pretreatment and hydrolysis. Ind Eng Chem Res..

[41] Seno ET, Chater KF (1983). Glycerol catabolic enzymes and their regulation in wild-type and mutant strains of *Streptomyces coelicolor* A3(2). J Gen Microbiol.

