# Supplementary material for: Pleiotropic regulation of a glucose-specific PTS in Clostridium acetobutylicum for high-efficient butanol production from corn stover without detoxification
Source: Biotechnol Biofuels. 2019 Nov 7;12:264. doi: 10.1186/s13068-019-1604-7 (PMC6836401; doi:10.1186/s13068-019-1604-7)
Supplement: Supplementary file 1 — Additional file 1: Table S1. Primers used in this study. [file 13068_2019_1604_MOESM1_ESM.docx]

**Table S1** Primers used in this study

| **Primers^a^** | **Sequence (5’→3’)** | **Description** |
| --- | --- | --- |
| glcG269\|270a–IBS | AAAACTCGAGATAATTATCCTTAGTATCCCCTGCTGTGCGCCCAGATAGGGTG | *glcG* Targetron primer |
| glcG269\|270a–EBS1d | CAGATTGTACAAATGTGGTGATAACAGATAAGTCCCTGCTGTTAACTTACCTTTCTTTGT | *glcG* Targetron primer |
| glcG269\|270a–EBS2 | TGAACGCAAGTTTCTAATTTCGATTGATACTCGATAGAGGAAAGTGTCT | *glcG* Targetron primer |
| EBS universal primer | CGAAATTAGAAACTTGCGTTCAGTAAAC | *glcG* Targetron primer |
| glcG­126-145 | TGTTCAAGCAGCAGGAAATG | forward primer inside *glcG* of 126 to 145 bp |
| glcG­473-492 | TCCACCAAAGAAACCCAAAA | reverse primer inside *glcG* of 473 to 492 bp |

^a^ as documented by Xiao et al. [11]
